# A Novel Link Between Electron Transport Chain Modulation and mcl‐PHA Production in *Pseudomonas aeruginosa* 5300 Using Azide as a Modulator

**DOI:** 10.1111/1758-2229.70251

**Published:** 2026-01-23

**Authors:** Raghavendra Paduvari, Divyashree Somashekara

**Affiliations:** ^1^ Department of Biotechnology Manipal Institute of Technology, Manipal Academy of Higher Education Manipal Karnataka India

**Keywords:** bioplastic, biopolymer, cytochrome, electron transport chain, heme‐copper oxidase, oxidative phosphorylation, polyhydroxyalkanoate

## Abstract

Medium chain length polyhydroxyalkanoates (mcl‐PHA) are elastomeric biodegradable bioplastics produced by a few bacteria. The large‐scale mcl‐PHA production remains limited by the low yield of bacterial strains and biomass reduction due to prolonged nutrient limitation in the media. In the present study, *Pseudomonas aeruginosa* MCC 5300 produced mcl‐PHA copolymer of about 39.4% cell dry weight (CDW) at a short duration of 24 h of growth in tryptic soy broth media containing oleic acid and the PHA content was enhanced up to 66.4% CDW in the presence of azide. The production of mcl‐PHA at such a short duration was not reported previously in nutrient‐enriched conditions. The application of azide enhanced mcl‐PHA production in the bacteria. Oleic acid shifted the electron transport chain (ETC) from the cytochrome c pool to the ubiquinol pool. The inhibition of bo_3_‐oxidase by azide increased electron flux towards bd‐oxidase to maintain proton gradient for oxidative phosphorylation, causing depletion of cellular reduced‐redox cofactor levels. The increased mcl‐PHA accumulation in bacteria compensated for the loss of reduced‐redox cofactors, thus maintaining cellular redox homeostasis. Hence, the study invokes a novel link between the ETC and mcl‐PHA production.

## Graphical abstract

The oleic acid and azide inhibited the cytochrome c oxidase activity in *Pseudomonas aeruginosa* MCC 5300, shifting the electron flux towards bd‐oxidase. This resulted in more NADH consumption to maintain the proton gradient for energy generation. The rapid depletion of NADH triggered high mcl‐PHA accumulation.

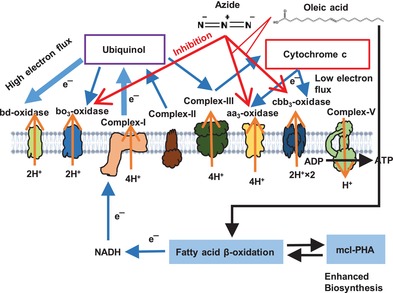

## 1 Introduction

The extensive use of petroleum‐based plastics in daily life has caused a severe negative impact on various ecosystems due to their non‐biodegradability and toxic nature. Even though the effects of these non‐biodegradable plastics are catastrophic to the environment over a long period, reducing their dependency is difficult due to their vast application. Polyhydroxyalkanoates (PHA) are bio‐based biodegradable plastics produced by bacteria that are an alternative to petroleum‐based plastics. PHA are polyesters of (*R*)‐3‐hydroxyalkanoic acid synthesised by bacteria as intracellular inclusions during physiological stress and nutrient‐deprived conditions (Raza et al. 2018). These biopolymers serve as an energy reserve for the bacterial cells during stress conditions, and they are produced when cultured in media containing excess carbon source and limitation of vital nutrients such as nitrogen, oxygen, phosphorus and magnesium (Kumar et al. 2020). The PHA is characterised by high tensile strength, durability, non‐toxic, biocompatible, water‐insoluble, resistance to hydrolytic damage and resistance to ultraviolet degradation. Unlike other petroleum‐based plastics, the PHA can be recycled (Muhammadi et al. 2015). Besides having similar and unique properties compared to petroleum‐based plastics, the PHA production on a large scale is very low. The low output is mainly due to the lower PHA yield of some bacterial strains and reduced biomass due to nutrient limitation and the reduced oxygen supply for accumulating large amounts of PHA.

The PHA are classified based on the number of carbon atoms in their monomers. They are short chain length (scl) PHA with 3–5 carbon atoms, medium chain length (mcl) PHA with 6–14 carbon atoms and long chain length (lcl) PHA with more than 14 carbon atoms (Kumar et al. 2020). The PHA polymer can be either homopolymers, composed of a single monomer or copolymers with multiple monomers (Li et al. 2016). The mcl‐PHA are synthesised by a few bacteria, such as *Pseudomonas* sp. and *Burkholderia* sp. mainly through fatty acid β‐oxidation or de novo fatty acid synthesis. These polymers have unique properties like reduced crystallinity, high tensile strength, low melting and glass transition temperatures, piezoelectric and elastomeric nature, making them suitable for industrial and biomedical applications such as in the preparation of body implants, biological latex and rubber‐like materials. They are used to make polymer scaffolds in tissue engineering and matrices for controlled drug delivery systems (Koller 2020; Silva et al. 2021; Tan et al. 2016). *Pseudomonas* sp. synthesise mcl‐PHA using class‐II PHA synthases, which allow the incorporation of diverse mcl‐PHA monomers based on the fatty acid chain length in the growth medium (Mozejko‐Ciesielska et al. 2019; Mohd Razaif‐Mazinah et al. 2016). Earlier studies using *Pseudomonas aeruginosa* MCC 5300 have shown that the bacterial growth in media containing oleic acid resulted in the production of mcl‐PHA copolymer containing higher chain lengths such as 3‐hydroxydecanoate and 3‐hydroxydodecanoate copolymers (Paduvari et al. 2024).

Bacterial respiration occurs via a series of enzymes called respiratory complexes, embedded in the plasma membrane, which form the electron transport chain (ETC). Reduced redox cofactors, such as nicotinamide adenine dinucleotide (NADH) and flavin adenine dinucleotide (FADH_2_) from different pathways donate electrons to the ETC. These electrons are sequentially transferred between respiratory complexes, ultimately transferring them to an oxygen molecule resulting in reduction to water (Melin et al. 2018). Terminal oxidase is a respiratory complex that reduces oxygen and terminates the ETC. These oxidase enzymes are cytochrome complexes that facilitate redox reactions (Richter and Ludwig 2009). The cytochromes are proteins comprising a heme group bound to the apoprotein (Cramer and Kallas 2016). The energy driven by electron transfer through these complexes is utilised in pumping protons from the bacterial cytoplasm to the periplasm, creating a transmembrane proton gradient. The kinetic energy of the proton backflow to the cytoplasm drives ATP synthase to produce ATP as cellular energy through oxidative phosphorylation (Ludwig et al. 2001). Oxygen limitation is known to be one of the stress factors that induce PHA production in bacteria (Madhusoodanan et al. 2021). Hence, a possible link might exist between terminal oxidase activity, the ETC and PHA production.

*P. aeruginosa* utilises a branched ETC that terminates with either cytochrome c oxidases or ubiquinol oxidases during aerobic growth. The bacterium has two isoforms each of type C (cbb_3_‐1 and cbb_3_‐2 oxidase) and type A (aa_3_‐oxidase) cytochrome c oxidases, along with two ubiquinol oxidases (bo_3_ and bd oxidase) (Arai 2011). Cytochrome c oxidases receive electrons from the cytochrome c pool. In contrast, ubiquinol oxidases receive them from the ubiquinol pool, with the bacterium switching between these electron donors based on environmental conditions (Kawakami et al. 2010). Due to various terminal oxidases and PHA production capability, the present study explores the link between terminal oxidase activity and PHA production in *P. aeruginosa* MCC 5300. The study aims to induce stress by inhibiting specific terminal oxidase activity in bacteria using azide ions (N_3_^−^) and enhance PHA production in nutrient‐enriched media conditions.

## 2 Materials and Methods

### 2.1 Bacterial Growth and Production of PHA

#### 2.1.1 Preparation of Bacterial Inoculum

The bacterial culture previously isolated from oil mill effluents and identified as *P. aeruginosa* MCC 5300 was used in the present study. A loopful of *P. aeruginosa* MCC 5300 colonies was transferred using a sterile inoculation loop to 50 mL nutrient broth media taken in a 250 mL Erlenmeyer flask under aseptic conditions and incubated at 30°C, 170 rpm for 18 h.

#### 2.1.2 Preparation of PHA Production Media and Bacterial Growth

The tryptic soy broth (TSB) media (M011‐500G, HiMedia) was prepared as per the manufacturer's instructions and 38 mM oleic acid was added as an additional carbon source. The media was sterilised using an autoclave. Sodium azide was added to the media before inoculation from the stock solution (65 mg/mL) to give appropriate final concentrations. 10 mL of bacterial inoculum was transferred into 100 mL TSB media in 500 mL Erlenmeyer flasks under aseptic conditions and incubated at 30°C, 170 rpm under shake‐flask conditions.

### 2.2 Bacterial Growth Analysis

10 mL of bacterial inoculum was transferred to 100 mL of PHA production media and incubated at 30°C, 170 rpm. Using a spectrophotometer, the bacterial growth was monitored using optical density measurements at a wavelength of 620 nm every 4 h of cultivation.

### 2.3 Estimation of Biomass

The bacterial cultures were harvested at regular intervals and centrifuged at 7781*g* for 8 min to obtain the cell pellets. The supernatant was discarded, and the cell pellets were washed with distilled water and acetone to remove residual fatty acid. The biomass was estimated gravimetrically (Katagi et al. 2023).

### 2.4 Extraction and Quantitative Estimation of PHA

The PHA was extracted from the bacterial biomass using the sodium hypochlorite–chloroform dispersion method. The PHA films obtained were washed with *n*‐hexane to remove residual lipids and fatty acids. The PHA was estimated gravimetrically (Rai et al. 2011).

### 2.5 Estimation of Cytochrome c Oxidase Activity

The bacterial cultures were centrifuged at 7741*g* for 8 min to obtain the cell pellets and washed twice with distilled water. The cell pellets were suspended in 50 mM phosphate buffer (pH = 6) to an optical density of 0.1 at 620 nm. The reaction was started by adding 4 μL of 50 mM *N*, *N*, *N*′, *N*′‐tetramethyl‐*p*‐phenylenediamine (TMPD) solution to 996 μL of the cell suspension and the absorbance changes at 610 nm were estimated. The rate of increase in the oxidised TMPD concentration was determined, and the cytochrome c oxidase activity (CcO activity) was found by subtracting the rate of TMPD autoxidation (Kassem et al. 2017).

### 2.6 Quantification of Cytoplasmic NAD(P)H

The cell pellets were washed in distilled water and suspended in 20 mM Tris–HCl buffer (pH = 8). The optical density at 620 nm was adjusted to 1. The cells were lysed by sonication and centrifuged at 9520*g* for 10 min at 4°C (Singh et al. 2008). The reduced‐redox cofactors (NADH and NADPH) were quantified in cytoplasmic fractions using fluorescence at 440 nm after 340 nm excitation (Wos and Pollard 2006).

### 2.7 Estimation of Residual Oleic Acid

The bacterial culture supernatant was mixed with equal volumes of isooctane to extract the oleic acid. The residual oleic acid was quantified using the spectrophotometric method by forming oleic acid copper soap in isooctane (Lowry and Tinsley 1976).

### 2.8 Characterisation of PHA Using FTIR and Proton‐NMR Spectroscopy

The infrared spectrum was obtained by directly analysing 5 mg of PHA using a Shimadzu FTIR‐based plastic analyser at 400–5000 cm^−1^ wavenumber. For proton NMR spectroscopy, 5 mg of PHA was dissolved in 1 mL of deuterated chloroform and analysed using Bruker Ascend 400 MHz NMR spectroscopy (Geethu et al. 2021).

### 2.9 Statistical Analysis

The experiments were performed as triplicates (*n* = 3) and plotted as an average of the three independent trials with the error bars indicating the standard deviation. The statistical significance was tested for the bacterial PHA production during azide inhibition in TSB containing oleic acid (TSBOA) using one‐way ANOVA, followed by Dunnett's multiple comparison post hoc analysis to find a significant PHA yield compared to the control.

## 3 Results and Discussion

### 3.1 *P. aeruginosa* MCC 5300 Cultured in TSB Media at Various Sodium Azide Concentrations

#### 3.1.1 Growth and PHA Production

**FIGURE 1:**
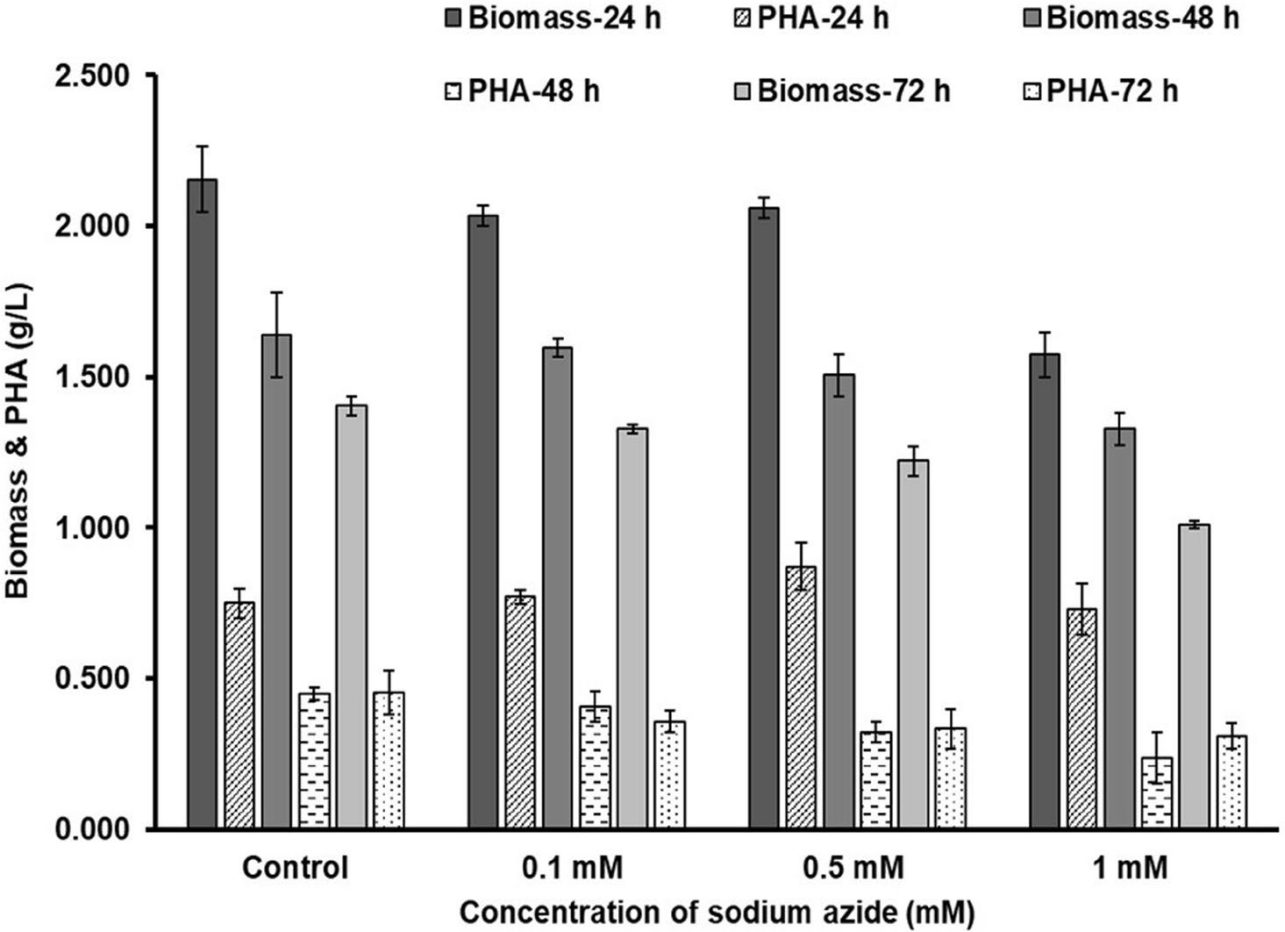
Growth and PHA production by *Pseudomonas aeruginosa* MCC 5300 in TSB media containing various concentrations of sodium azide at 30°C, 170 rpm for 24, 48 and 72 h of incubation by taking TSB media without sodium azide as a control.

*P. aeruginosa* MCC 5300 was cultured in TSB media at sodium azide concentrations of 0.1, 0.5 and 1 mM along with TSB without sodium azide as a control (Figure 1). The concentration range below the minimum inhibitory concentration of 3 mM was selected for the study based on the preliminary experiment done in the lab (Figure S1). A biomass of 2.036 and 2.061 g/L was observed at sodium azide concentrations of 0.1 and 0.5 mM, respectively, similar to the control with a biomass of 2.155 g/L but an increase in concentration to 1 mM caused a 1.36‐fold reduction with a biomass of 1.575 g/L. A significant decrease in the biomass was observed in all the conditions at 48 and 72 h of incubation. The biomass remained low at a 1 mM sodium azide concentration compared to the lower concentrations and control at all time points.

A sodium azide concentration of 0.1, 0.5 and 1 mM produced a PHA yield of about 0.771, 0.872 and 0.729 g/L, respectively, compared to the control with a PHA yield of 0.749 g/L in bacteria. There was no significant change in the PHA yield across various concentrations of sodium azide and the yield was similar to the controls. Hence, the presence of azide did not cause changes in the PHA yield at 24 h of incubation. A prolonged incubation to 48 and 72 h of incubation significantly reduced the PHA yield at all growth conditions (Figure 1). This is due to the intracellular degradation of the PHA polymer by PHA depolymerase into (*R*)‐3‐hydroxyalkanoate monomers, which generates energy upon mobilisation to sustain bacterial growth (Solaiman et al. 2003).

#### 3.1.2 CcO Activity

**FIGURE 2:**
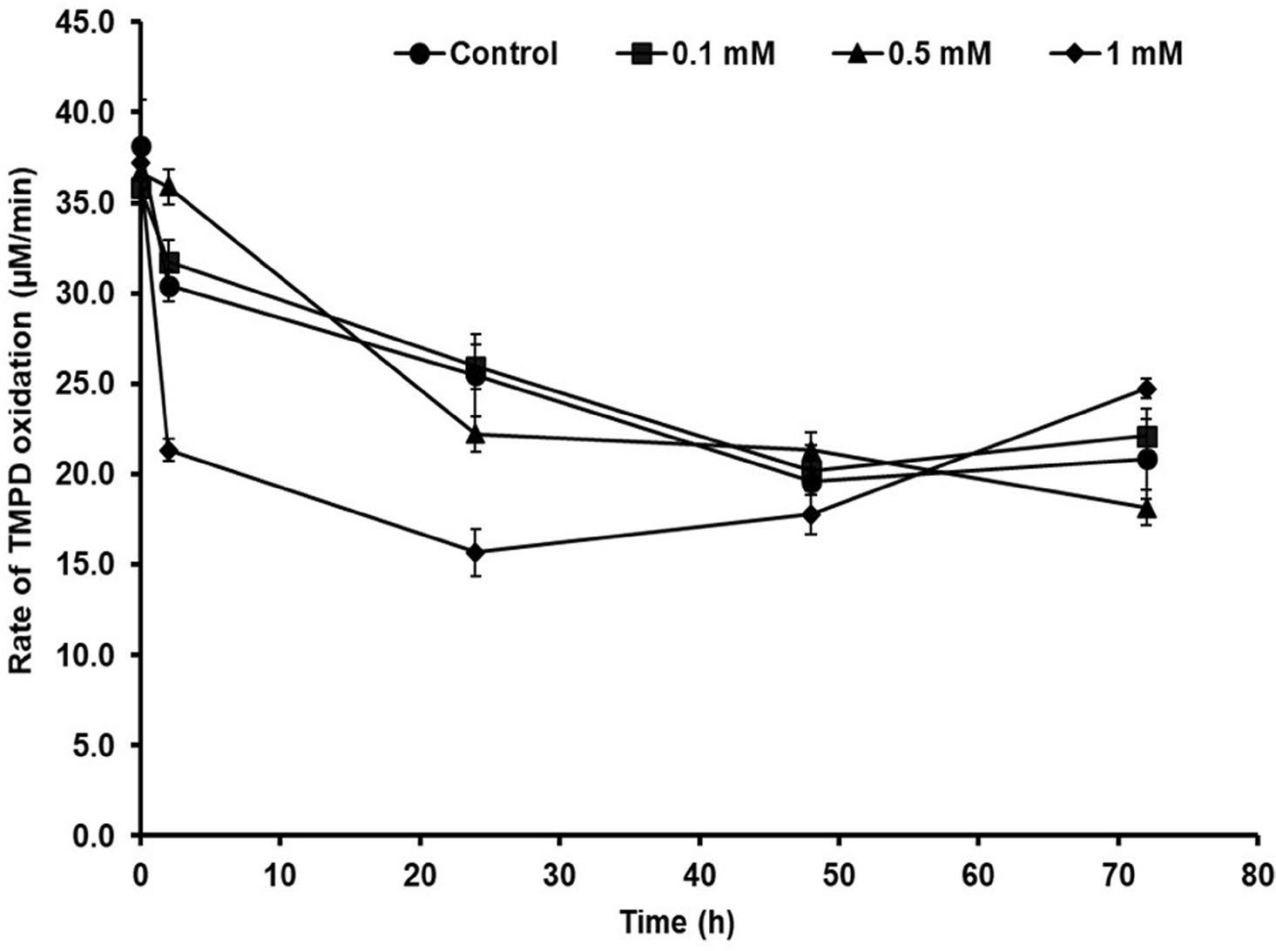
Activity of cytochrome c oxidases in *Pseudomonas aeruginosa* MCC 5300 cultured in TSB media containing various sodium azide (mM) concentrations at 30°C, 170 rpm.

*P. aeruginosa* MCC 5300 cultures showed an initial CcO activity of 35.8, 36.7 and 37.2 μM/min at sodium azide concentrations of 0.1, 0.5 and 1 mM, respectively, similar to the control with an activity of 38.2 μM/min. Sodium azide concentrations of 0.1 and 0.5 mM resulted in CcO activities of 31.7 and 35.9 μM/min, respectively, similar to the control with an activity of 30.4 μM/min at 2 h of incubation but an increase in the concentration to 1 mM caused a significant reduction in the activity to 21 μM/min. A 1.6‐fold decrease in the activity of about 15.7 μM/min was observed at 1 mM concentration at 24 h of incubation compared to the control with an activity of 25.5 μM/min. Low concentrations of 0.1 and 0.5 mM did not cause significant changes in the activity. The CcO activity at different sodium azide concentrations was similar to the control at 48 h of growth and it remained unchanged till 72 h (Figure 2).

The studies conducted on *Paracoccus denitrificans* have shown that the azide ion is a potent inhibitor of cytochrome c oxidases that coordinately bind to the heme a_3_ iron and Cu_B_ copper atom at the active site, forming an interatomic molecular bridge and disrupting the enzyme activity (Vamvouka et al. 1999). The previous studies conducted on *P. putida* F1 showed a similar effect of azide on cytochrome c oxidases and inhibited aerobic growth (Lai et al. 2020). In the present study on *P. aeruginosa* MCC 5300, inhibition of CcO activity by azide did not cause any changes in the PHA yield, and it remained constant at increasing concentrations of azide. An increase in sodium azide concentration to 0.5 mM did not cause any changes in the CcO activity or biomass. Still, a concentration of 1 mM reduced the CcO activity due to the azide inhibition, causing a decrease in the biomass (Figures 1 and 2).

### 3.2 *P. aeruginosa* MCC 5300 Cultured in TSB Media Containing Oleic Acid at Various Sodium Azide Concentrations

#### 3.2.1 Growth and PHA Production

**FIGURE 3:**
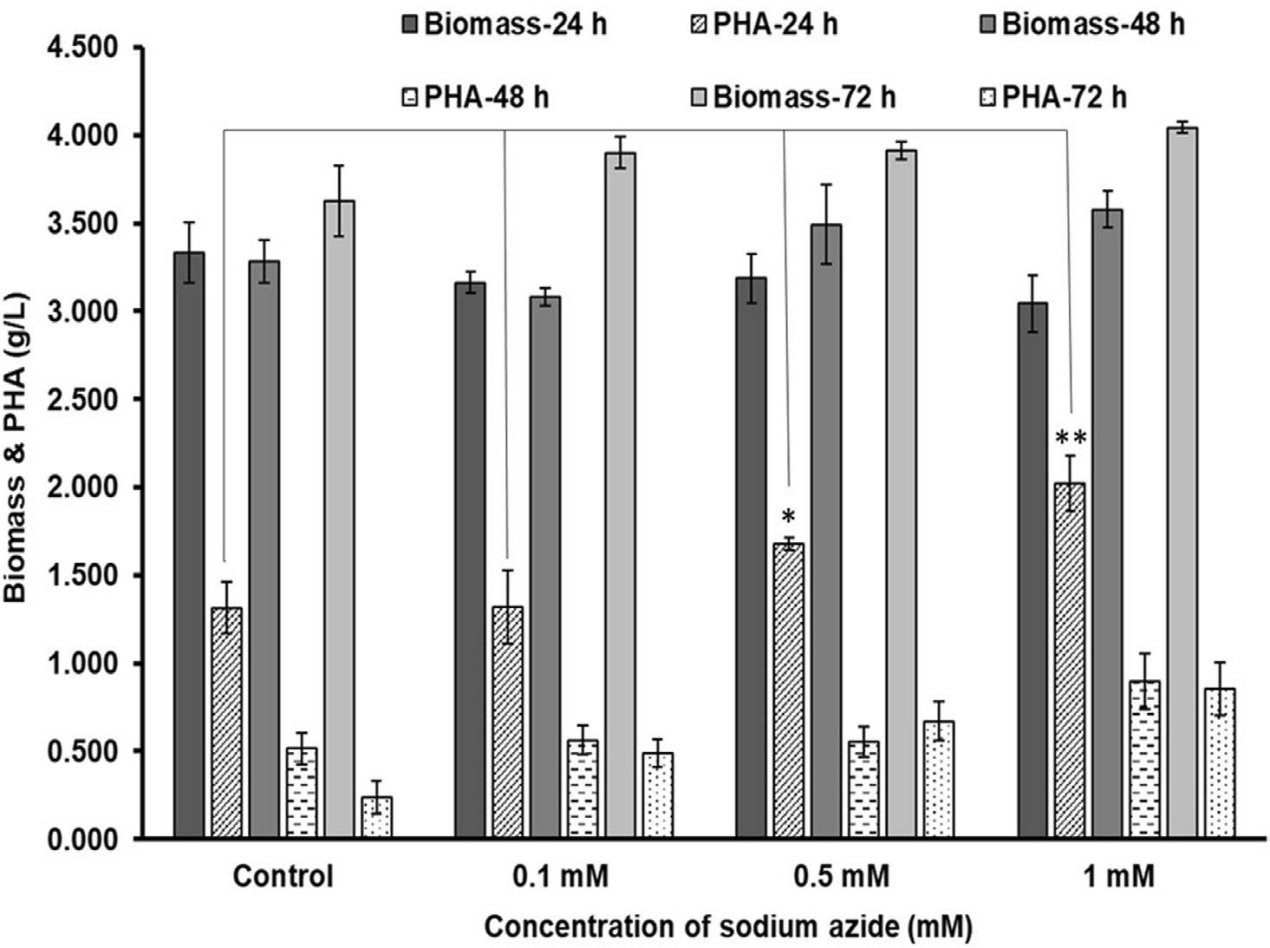
Growth and PHA production by *Pseudomonas aeruginosa* MCC 5300 in TSB media containing 38 mM oleic acid at different concentrations of sodium azide at 30°C, 170 rpm for 24, 48 and 72 h of incubation by taking TSB media containing only oleic acid as a control. A one‐way ANOVA followed by Dunnett's multiple comparison post hoc test was used to determine the significant increase in PHA yield. *Significant (*p* < 0.05) PHA yield increase in 0.5 and 1 mM sodium azide concentrations compared to the control.

*P. aeruginosa* MCC 5300 was analysed for growth and PHA production in TSB media containing oleic acid as a carbon source and sodium azide at a concentration of 0.1, 0.5 and 1 mM along with TSB media containing only oleic acid as a control (Figure 3). The presence of oleic acid in TSB media caused an increase in biomass at all the conditions of growth compared to TSB media devoid of oleic acid. The increased biomass indicates that the bacterial cells effectively utilise the oleic acid for growth (Figures 1 and 3). The bacteria grew to a biomass of 3.161, 3.187 and 3.043 g/L at a sodium azide concentration of 0.1, 0.5 and 1 mM, respectively, similar to the control with a biomass of 3.335 g/L at 24 h of incubation (Figure 3). The biomass remained constant across different concentrations of sodium azide. It did not cause a reduction at 1 mM concentration as in the case of TSB media devoid of oleic acid. The biomass remained nearly constant at 48 h of incubation at all the concentrations with a biomass of 3.079, 3.491 and 3.577 g/L at 0.1, 0.5 and 1 mM, respectively, similar to the control with a biomass of 3.28 g/L. An increase in biomass of about 3.901, 3.912 and 4.044 g/L was observed after 72 h of incubation at a sodium azide concentration of 0.1, 0.5 and 1 mM, respectively. The biomass increased at successive time points of growth in the presence of oleic acid and sodium azide compared to a decrease in biomass observed in the absence of oleic acid (Figures 1 and 3).

The sodium azide at a concentration of 0.1 mM in the media produced a PHA yield of about 1.319 g/L with a PHA content of 41.7% cell dry weight (CDW), similar to the control with a PHA yield of 1.316 g/L and a PHA content of 39.4% CDW at 24 h of incubation. An increase in the concentration of sodium azide to 0.5 mM caused a 1.27‐fold increase in the PHA yield to 1.677 g/L with a PHA content of 52.6% CDW compared to the control at 24 h. Further, an increase in the concentration to 1 mM enhanced PHA production up to 1.53‐fold with a PHA yield of 2.021 g/L and a PHA content of 66.4% CDW compared to the control at 24 h. The one‐way ANOVA of the control and the test samples showed that an increase in the concentration of sodium azide significantly increased the PHA production with a *p*‐value of 0.0012 (*p* < 0.05). Further analysis using Dunnett's multiple comparison test showed that a sodium azide concentration of 0.5 and 1 mM caused a significant increase in the PHA yield with a *p*‐value of 0.046 and 0.0011, respectively, at 24 h of growth. A concentration of 0.1 mM did not cause a significant difference in PHA yield, with a *p*‐value of 0.999 above 0.05. A gradual reduction in PHA was observed at all the conditions of growth at 48 and 72 h of incubation. The PHA yield reached 0.561 and 0.553 g/L at a sodium azide concentration of 0.1 and 0.5 mM, respectively, similar to the control with a PHA yield of 0.515 g/L but an increase in concentration to 1 mM resulted in a slightly high PHA yield of about 0.9 g/L. Further incubation to 72 h resulted in a high PHA yield of about 0.492, 0.671 and 0.856 g/L at a sodium azide concentration of 0.1, 0.5 and 1 mM, respectively, compared to the control with a PHA yield of 0.237 g/L. An increase in sodium azide concentration in TSBOA improved the PHA yield in bacteria. The enhanced PHA yield in the presence of oleic acid compared to media devoid of oleic acid shows that *P. aeruginosa* MCC 5300 utilises oleic acid for PHA production and subsequent addition of sodium azide induces stress in the media, causing the diversion of oleic acid metabolites towards PHA production, thus increasing the volumetric PHA yield. The PHA were degraded and utilised by the bacterial cells for growth, causing an increase in biomass and a decrease in PHA yield at a prolonged duration (Figure 3). On the other hand, the bacteria grown in TSB media containing sodium azide devoid of oleic acid showed a decrease in biomass at prolonged incubation due to less PHA in the bacterial cells for growth and nourishment (Figure 1).

The PHA production was demonstrated in *P. aeruginosa* PAO1 cultured in mineral salt media (MSM) containing decanoic acid (C10) as a carbon source. The bacterium achieved a maximum PHA production of 9.7% CDW after 48 h under nutrient limitation (Chan et al. 2006). *P. putida* Bet001 produced PHA of about 11.4% CDW using oleic acid (C18) in MSM after a long duration of 48 h (Mohd Razaif‐Mazinah et al. 2016). *P. putida* LS46 produced PHA of 16.5% CDW using octanoic acid in MSM after 24 h under non‐sterile conditions (Blunt et al. 2024). However, in the present study involving *P. aeruginosa* MCC 5300, a high PHA content of 39.4% CDW was achieved at a very short duration of 24 h of growth compared to *P. aeruginosa* PAO1 and *P. putida* Bet001, which took 48 h. The PHA content reached a maximum value in *P. putida* LS46 at 24 h. Still, it was 2.3‐fold lower than the PHA content of *P. aeruginosa* MCC 5300. The *P. aeruginosa* MCC 5300 produced high PHA in nutrient‐enriched conditions in contrast to nutrient‐limiting conditions in *P. aeruginosa* PAO1, *P. putida* Bet001 and *P. putida* LS46. Further, the azide ions increased the PHA content in the bacterial cells up to 66.4% CDW at 24 h of growth (Figure 3). It surpassed the PHA content of 32.07% CDW, 22.57% CDW and 35.93% CDW by *P. putida* KT2440 using sodium salts of fatty acid laurate (C12), tridecanoate (C13) and myristate (C14) after 68 h of incubation (Sikkema et al. 2023). Hence, this is the first report of using azide‐induced stress to enhance PHA production in bacteria in nutrient‐enriched media without growth inhibition and nutrient limitation.

The PHA production in bacteria requires nutrient limitation, reduced aeration and limited oxygen concentration in the media. Therefore, a prolonged incubation time in these conditions has become a norm in inducing PHA accumulation and achieving maximum production in bacteria, but it significantly reduces the biomass. The reduced biomass due to stress conditions causes a decrease in PHA yield (Pratt et al. 2012). The high PHA production in *P. aeruginosa* MCC 5300 at a short duration in the nutrient‐enriched media evades the biomass reduction during nutrient limitation. Further, enhancement of PHA yield by azide below its growth inhibitory concentration will allow bacterial growth due to the absence of nutrient limitation and improve industrial PHA production. The negative effect of prolonged oxygen limitation on bacterial growth can be avoided, and the maintenance cost for controlling oxygen concentration can be reduced on an industrial scale.

#### 3.2.2 Cytochrome c Oxidase Activity

**FIGURE 4:**
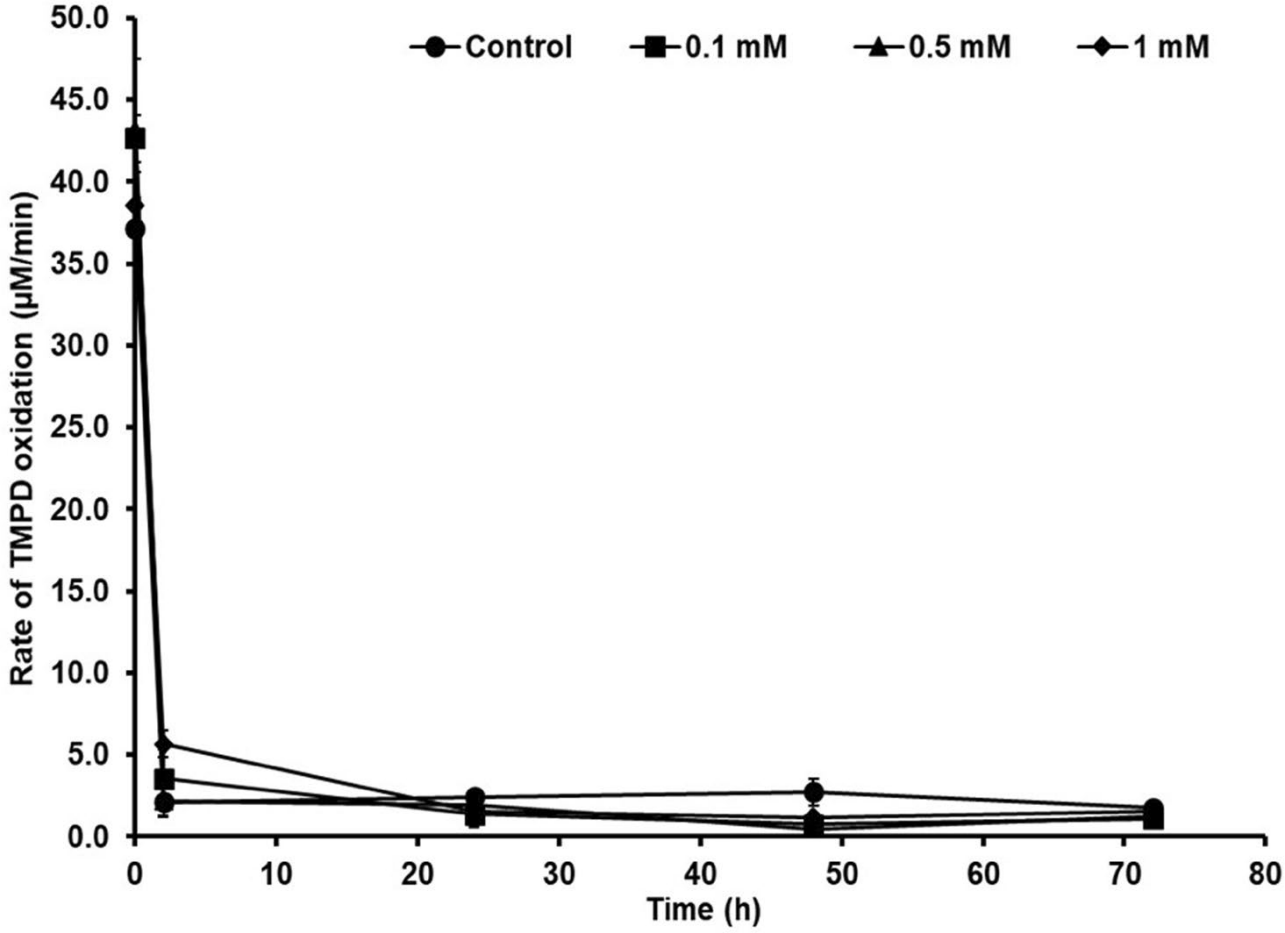
Activity of cytochrome c oxidases in *Pseudomonas aeruginosa* MCC 5300 cultured in TSB media containing 38 mM oleic acid and sodium azide at various concentrations (mM) at 30°C, 170 rpm.

*P. aeruginosa* MCC 5300 showed an initial CcO activity of 42.6, 43.2 and 38.6 μM/min in TSB containing 0.1, 0.5 and 1 mM sodium azide concentrations, respectively, which was similar to the control with an activity of 37.1 μM/min. A drastic decrease in the activity of about 3.5, 2.2 and 5.7 μM/min was observed at sodium azide concentrations of 0.1, 0.5 and 1 mM, respectively, at 2 h of incubation. The decrease in the CcO activity in the presence of sodium azide was similar to the control group, which showed less activity of about 2.1 μM/min at 2 h of incubation. The activity remained low at 24 h of incubation in the 0.5–2.7 μM/min range in bacteria cultured at all conditions containing oleic acid. It remained unchanged throughout incubation (Figure 4).

The presence of oleic acid in the media caused a decrease in the activity of cytochrome c oxidases in *P. aeruginosa* MCC 5300 at an early stage of incubation of about 2 h of growth. The reduced CcO activity in the bacteria was followed by a high volumetric PHA yield at 24 h of incubation. The addition of azide into the media improved the PHA yield and PHA content in the bacterial cells without growth inhibition and the change in concentration did not cause any changes in the CcO activity (Figures 3 and 4). An increase in PHA yield after a subsequent decrease in the CcO activity was observed only in the presence of oleic acid and absent in media without oleic acid (Figures 1 and 2).

The studies conducted on mutant *P. putida* KT2440 lacking the bo_3_‐oxidase have shown a significant upregulation in the bd‐oxidase and cbb_3_‐1 oxidase in the bacterium. It indicates that the terminal oxidases in *Pseudomonas* sp. are differentially expressed based on the growth condition in the media. Therefore, the absence of one terminal oxidase is compensated for by the other to generate optimum energy through oxidative phosphorylation (Morales et al. 2006). The lcl fatty acids such as palmitic acid, linoleic acid and oleic acid are known to inhibit the bovine heart CcO activity by interacting with the active site (Sharpe et al. 1997). The conserved fatty acid binding site was previously observed in the cytochrome c oxidase of *Rhodobacter sphaeroides* (Hiser et al. 2013). However, little is known about the inhibition of cytochrome c oxidases in *P. aeruginosa* . Hence, two different possibilities exist for reducing CcO activity by oleic acid. One possibility is through direct enzyme inhibition after oleic acid binds to the active site, and the other is through downregulation of cytochrome c oxidase gene expression in *P. aeruginosa* MCC 5300. The cytochrome c oxidase inhibition or downregulation shifts the ETC from the cytochrome c pool to the ubiquinol pool, causing an increase in the activity of ubiquinol oxidases. The ubiquinol oxidases will contribute to transmembrane proton gradient formation and energy generation through oxidative phosphorylation. Therefore, a reduced activity was observed after 2 h of incubation without affecting the bacterial growth. The azide in the media did not cause any changes in bacterial growth and CcO activity (Figures 3 and 4).

### 3.3 Bacterial Growth Analysis

**FIGURE 5:**
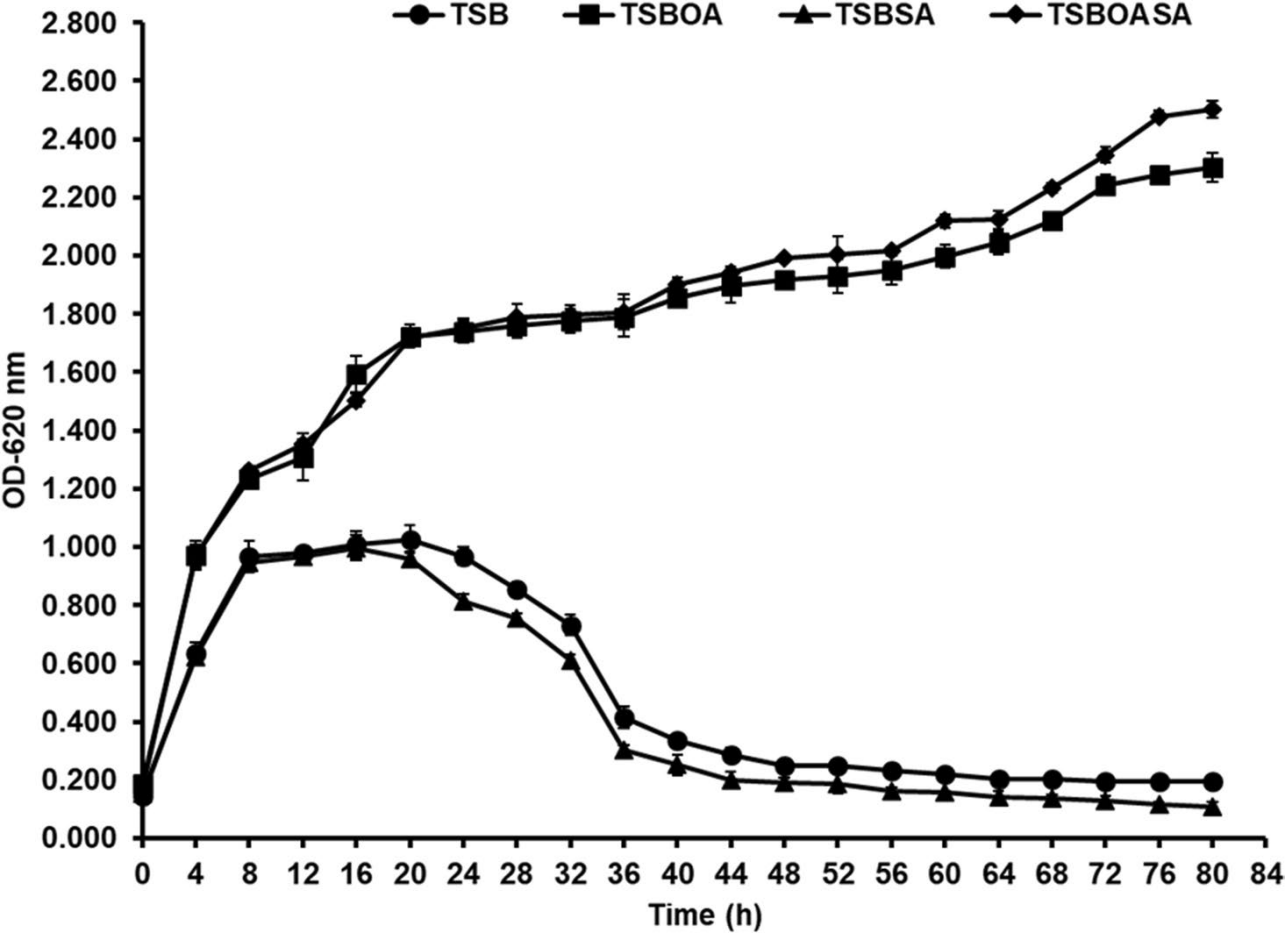
Bacterial growth analysis in TSB containing 1 mM sodium azide (TSBSA), TSB containing oleic acid (TSBOA) and TSB containing oleic acid and 1 mM sodium azide (TSBOASA) using only TSB media as a control. The cultures were incubated at 30°C, 170 rpm.

The bacterial growth was analyzed in *P. aeruginosa* MCC 5300 cultured in TSB media containing 1 mM sodium azide (TSBSA), and 1 mM sodium azide (TSBOASA) using only TSB media as a control. The optimum sodium azide concentration of 1 mM was selected for this study as the bacteria produced maximum PHA yield without inhibition of growth. The bacterial cultures in TSBSA showed three distinct growth phases similar to the control group. An exponential phase was marked by an increase in bacterial growth till 8 h of incubation, and a stationary phase was observed till 20 h of cultivation in TSBSA. A death phase was observed in TSBSA, marked by a decrease in bacterial growth after 20 h of incubation (Figure 5). A slight reduction in growth can be observed in TSBSA compared to the control group, which correlates with a slight decrease in the biomass at 1 mM sodium azide concentration (Figures 1 and 5).

The bacterial growth was higher in TSBOA and TSBOASA than in the control group. A steady increase in the growth was observed in the presence of oleic acid until 80 h of incubation, in contrast to TSBSA and the control. TSBOASA showed a slight increase in growth at 72 h compared to TSBOA, which correlates with the high biomass obtained in TSB media containing oleic acid and 1 mM sodium azide concentration (Figures 3 and 5).

### 3.4 Analysis of Intracellular Reduced‐Redox Cofactors and Oleic Acid Depletion During PHA Production in *P. aeruginosa* MCC 5300

**FIGURE 6:**
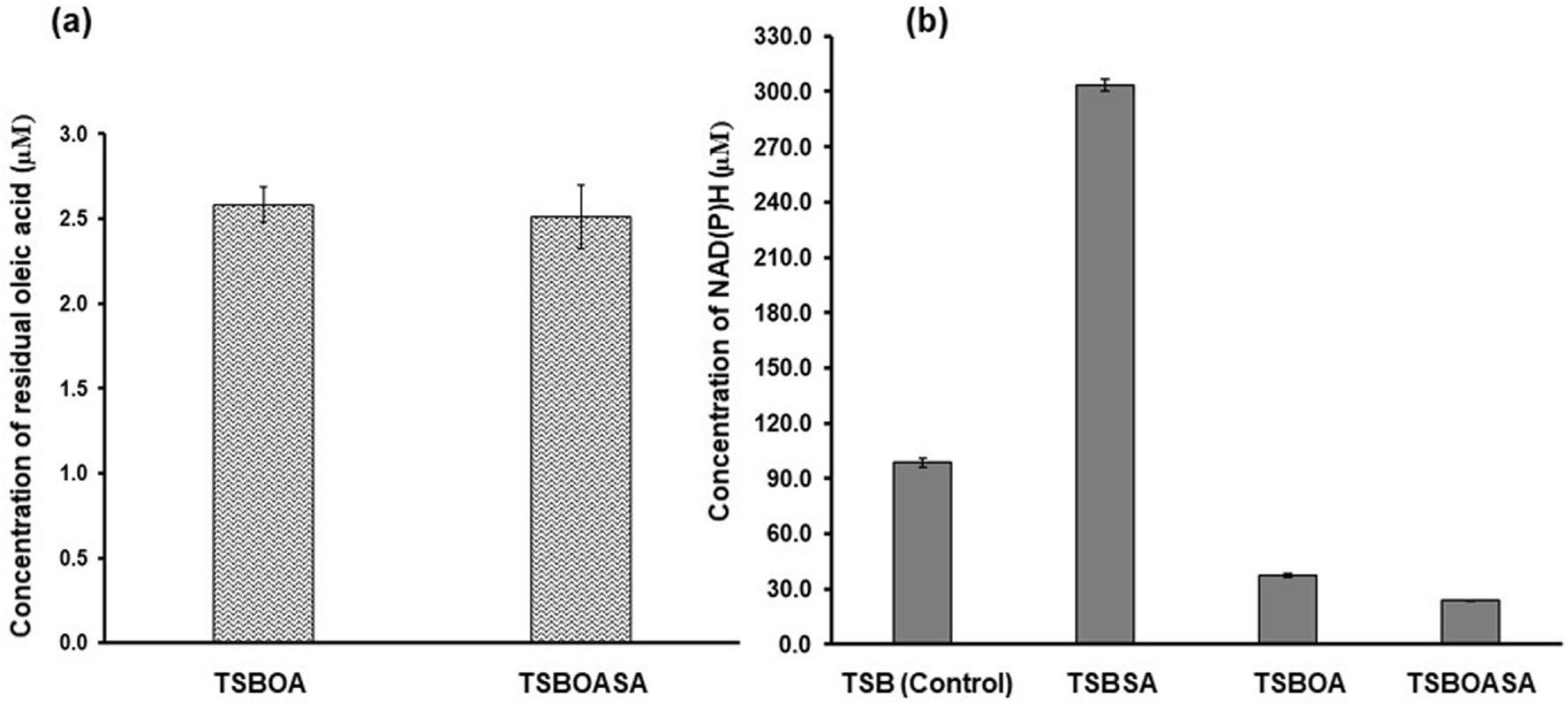
(a) Oleic acid depletion in *Pseudomonas aeruginosa* MCC 5300 cultured in different media conditions at 24 h of incubation. (b) Intracellular total reduced NADH and NADPH levels in *P. aeruginosa* MCC 5300 cultured in various conditions at 24 h of incubation.

The intracellular total reduced‐redox (NADH and NADPH) cofactors levels and residual oleic acid were analysed in *P. aeruginosa* MCC 5300 cultured in TSB media containing 1 mM sodium azide (TSBSA), TSBOA and TSB media containing oleic acid with 1 mM sodium azide (TSBOASA) along with TSB media devoid of oleic acid and sodium azide (TSB) as a control. A high depletion of oleic acid was observed at all the media conditions. A residual oleic acid concentration of 2.6 μM in TSBOASA similar to TSBOA with a concentration of 2.5 μM indicates that the oleic acid depletion rate remained unchanged despite changes in media conditions (Figure 6a). A threefold increase in the concentration of reduced‐redox cofactors was observed in TSBSA with a concentration of 303.7 μM compared to the control with a concentration of 98.7 μM. A 2.6‐fold reduction in reduced‐redox cofactors was observed in TSBOA with a concentration of 37.3 μM compared to the control and further reduced to 23.8 μM in TSBOASA (Figure 6b).

A study involving recombinant *P. aeruginosa* PAO6049 without genes encoding cyanide‐insensitive bd‐oxidase in its genome has shown that the bacterium could not grow in the presence of 1 mM sodium azide. This is due to the binding of the azide ion to bo_3_‐oxidase and other cytochrome c oxidases in *P. aeruginosa* PAO6049, but the wild‐type bacterium, consisting of bd‐oxidase genes, showed abundant growth. This indicates that the bd‐oxidase is essential during growth under cytochrome c oxidase inhibition (Cunningham and Williams 1995). The study conducted on azide inhibition of various terminal oxidases in *P. aeruginosa* PAO1 has shown that azide not only inhibits cytochrome c oxidases in the bacterium but also bo_3_‐oxidase that utilises ubiquinol as an electron donor. This is mainly due to the characteristic resemblance of bo_3_‐oxidase to cytochrome c oxidases since they belong to the same superfamily of proteins known as heme‐copper oxidase (HCO) (Hijazi et al. 2017). The HCO have a characteristic binuclear heme‐copper active site for oxygen reduction in contrast to bd‐oxidase, with an active site consisting of heme b and heme d (Arai 2011; Borisov et al. 2011). Due to the similarity of the active site, the azide ion binds and inhibits bo_3_‐oxidase activity. In the present study, the inhibition of HCO by azide caused a threefold high reduced‐redox cofactor level in the bacteria cultured in media in the presence of sodium azide without oleic acid (Figure 6b). The intracellular level of NADH is maintained at a higher level than NADPH in the bacteria (Xu et al. 2021). Therefore, the excess intracellular reduced‐redox cofactors can be attributed to high NADH accumulation in the cytoplasm due to inhibition of HCO. Since PHA accumulation was reduced due to the absence of oleic acid, the excess NADH was not stored as PHA in the bacteria, causing its accumulation in the cytoplasm, thus disrupting cellular redox homeostasis.

**FIGURE 7:**
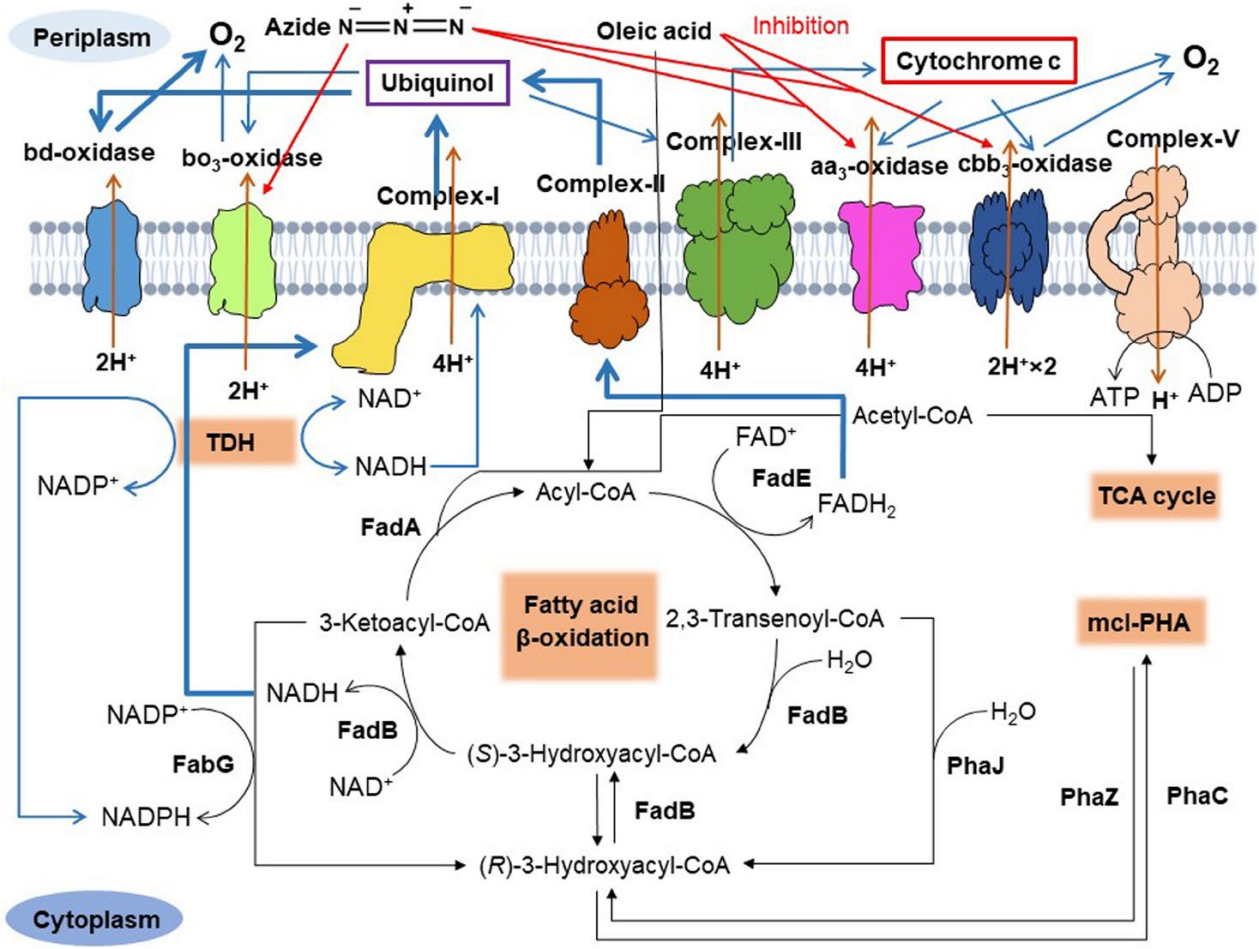
Illustration showing the electron transport chain in the presence of oleic acid and azide ion. The heavy blue arrows represent the high influx of electrons, light blue arrows show a low influx of electrons and proton transport is indicated by orange arrows. Complex‐I (NADH dehydrogenase), Complex‐II (Succinate dehydrogenase), Complex‐III (Cytochrome bc_1_ complex), Complex‐V (ATP synthase), FadA (3‐ketoacyl‐CoA thiolase), FadB (enoyl‐CoA hydratase/(*S*)‐3‐hydroxyacyl‐CoA reductase), FadE (acyl‐CoA dehydrogenase), TDH (transhydrogenase), PhaJ ((*R*)*‐*specific enoyl‐CoA hydratase), FabG (3‐ketoacyl‐CoA reductase), PhaC (PHA synthase) and PhaZ (PHA depolymerase).

The NADH originating from the fatty acid β‐oxidation pathway transfers electrons to NADH dehydrogenase (Complex‐I) and gets oxidised to NAD^+^. When the bacteria use cytochrome c oxidases, the electrons from Complex‐I are transferred to cytochrome bc_1_ complex (Complex‐III) via the ubiquinol molecule. The ubiquinol molecule also receives electrons from succinate dehydrogenase (Complex‐II) and other membrane‐bound dehydrogenases. The electrons from Complex‐III are transferred to cytochrome c oxidases via the cytochrome c molecule. Similarly, when the bacteria use ubiquinol oxidases, the electrons are directly transferred from the ubiquinol molecule to ubiquinol oxidases (Kawakami et al. 2010). The proton gradient generated by these complexes is utilised by ATP synthase (Complex‐IV) to generate ATP as cellular energy (Ciprich et al. 2022). Earlier studies have shown that the ETC terminated by cytochrome c oxidases produces more cellular energy than ubiquinol oxidases, mainly due to the high protons pumped into the periplasm to generate a proton gradient (Arai et al. 2014). In the present study, oleic acid‐induced reduction of cytochrome c oxidases diverted the electron flux from the cytochrome c pool to the ubiquinol pool. In order to compensate for the loss of cellular energy generation for bacterial growth, a high amount of electrons is transferred to ubiquinol oxidases, thus increasing the number of protons pumped into the periplasm. The high influx of electrons to ubiquinol oxidases caused a reduction in the intracellular level of reduced‐redox cofactors up to 2.6‐fold in the presence of oleic acid compared to the control. To prevent the depletion of NADH, PHA is accumulated at a higher amount in the bacterial cells, which, upon degradation by PHA depolymerase, restores the NADH and NADPH levels in the bacteria to sustain growth. A further reduction in reduced‐redox cofactors of about 4.1‐fold was observed in the presence of oleic acid and azide compared to the control. This is mainly due to bo_3_‐oxidase inhibition by azide ions, which leads to a high electron influx towards bd‐oxidase. The elevated activity of azide‐resistant bd‐oxidase rapidly drained cellular NADH levels for proton pumping and energy generation. The rapid depletion of cellular NADH during azide inhibition of HCO was compensated by enhanced accumulation of PHA in the bacteria, allowing it to grow and produce high PHA yield (Figures 6b and 7).

### 3.5 Characterisation of the Polymers Using FTIR Spectroscopy

The polymer produced by *P. aeruginosa* MCC 5300 in TSB media containing oleic acid and 1 mM sodium azide concentration was analysed using FTIR spectroscopy along with TSB media containing only oleic acid and polyhydroxybutyrate‐co‐valerate (PHBV) copolymer as control and standard, respectively. The infrared spectrum of the polymer showed 5 prominent peaks characteristic of the PHA. The peaks in a range of 3395–3442 cm^−1^ and 1719^1^–1722 cm^−1^ in the polymer indicate the stretching vibrations of the hydroxyl group (–OH) and carbonyl group (–C═O) similar to the standard PHBV. The stretching vibration observed at 2921–2933 cm^−1^ and 2852–2879 cm^−1^ indicates the stretching vibrations of methylene (–CH_2_) and –CH groups, respectively. A peak in the range of 1444–1453 cm^−1^ indicates the deformation vibration of methylene groups. The standard's hydroxyl, carbonyl, methylene and –CH groups form the essential backbone structure of the PHA polymer. The resemblance of peaks obtained for the polymer produced by *P. aeruginosa* MCC 5300 indicates it is a PHA. No changes were observed among the prominent peaks in the PHA polymer produced in the presence of azide compared to the control. A notable peak for the methylene group stretch in the PHA compared to the standard PHBV indicates the presence of multiple methylene groups attached to the PHA backbone (Figures S2, 8 and 9). In a previous study, a similar peak was observed at 2924 cm^−1^ in mcl‐PHA obtained from *P. aeruginosa* (Tanikkul et al. 2020). This indicates that the polymer obtained from *P. aeruginosa* MCC 5300 in the present study is a mcl‐PHA.

**FIGURE 8:**
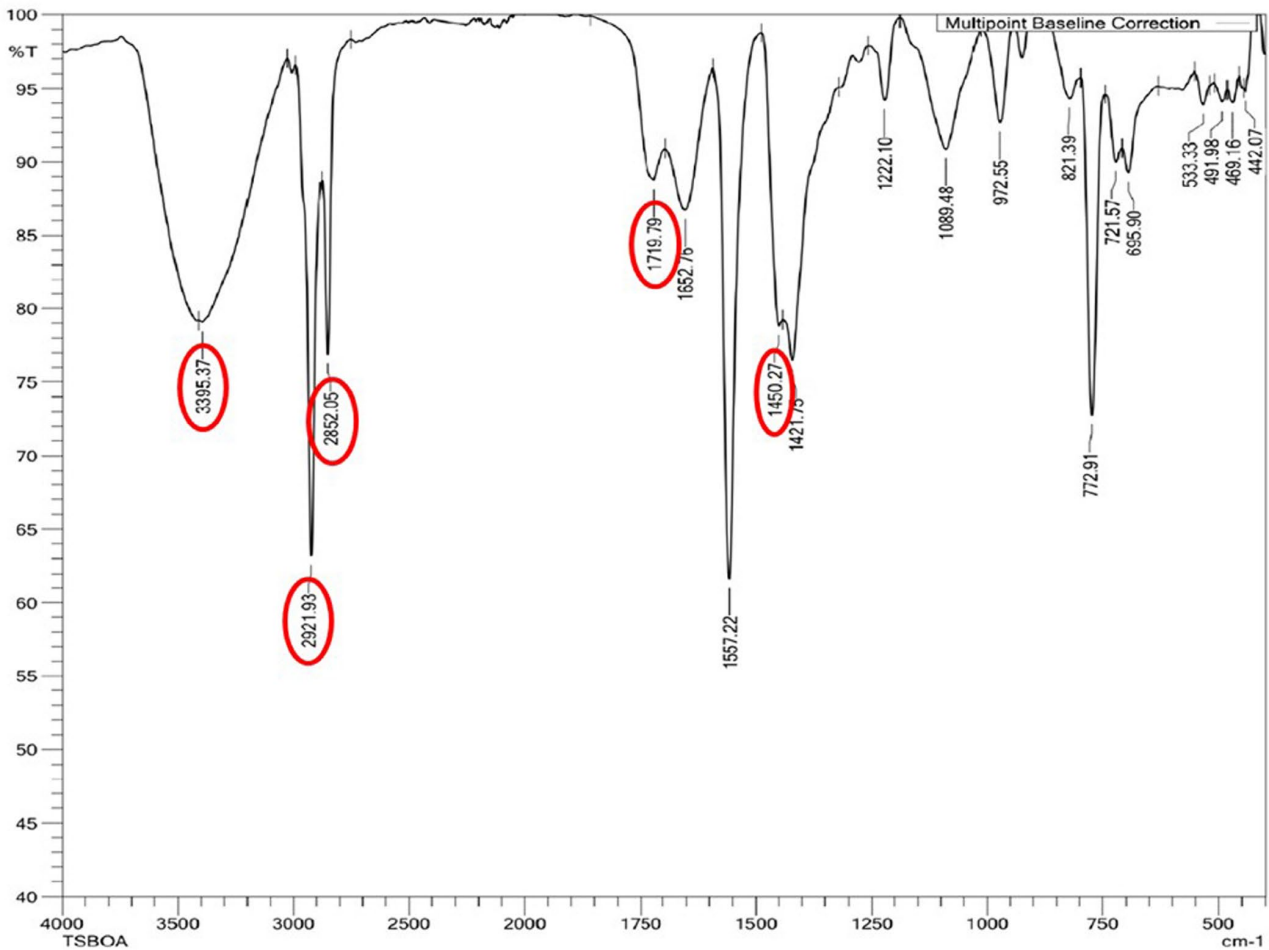
FTIR spectroscopic analysis of polymer produced by *Pseudomonas aeruginosa* MCC 5300 in TSB media containing oleic acid.

**FIGURE 9:**
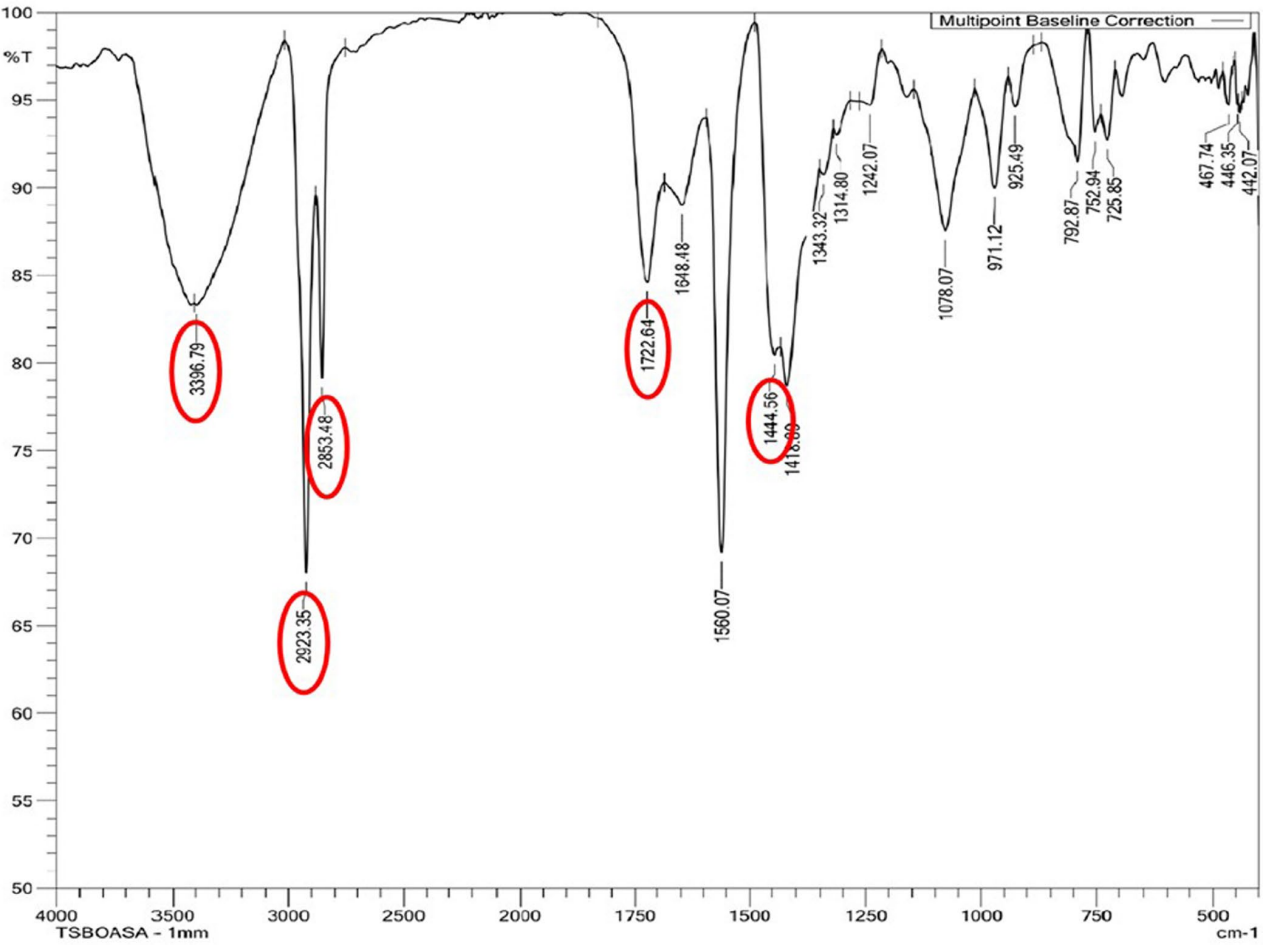
FTIR spectroscopic analysis of polymer produced by *Pseudomonas aeruginosa* MCC 5300 in TSB media containing oleic acid and 1 mM sodium azide.

### 3.6 Characterisation of the Polymers Using Proton NMR Spectroscopy

**FIGURE 10:**
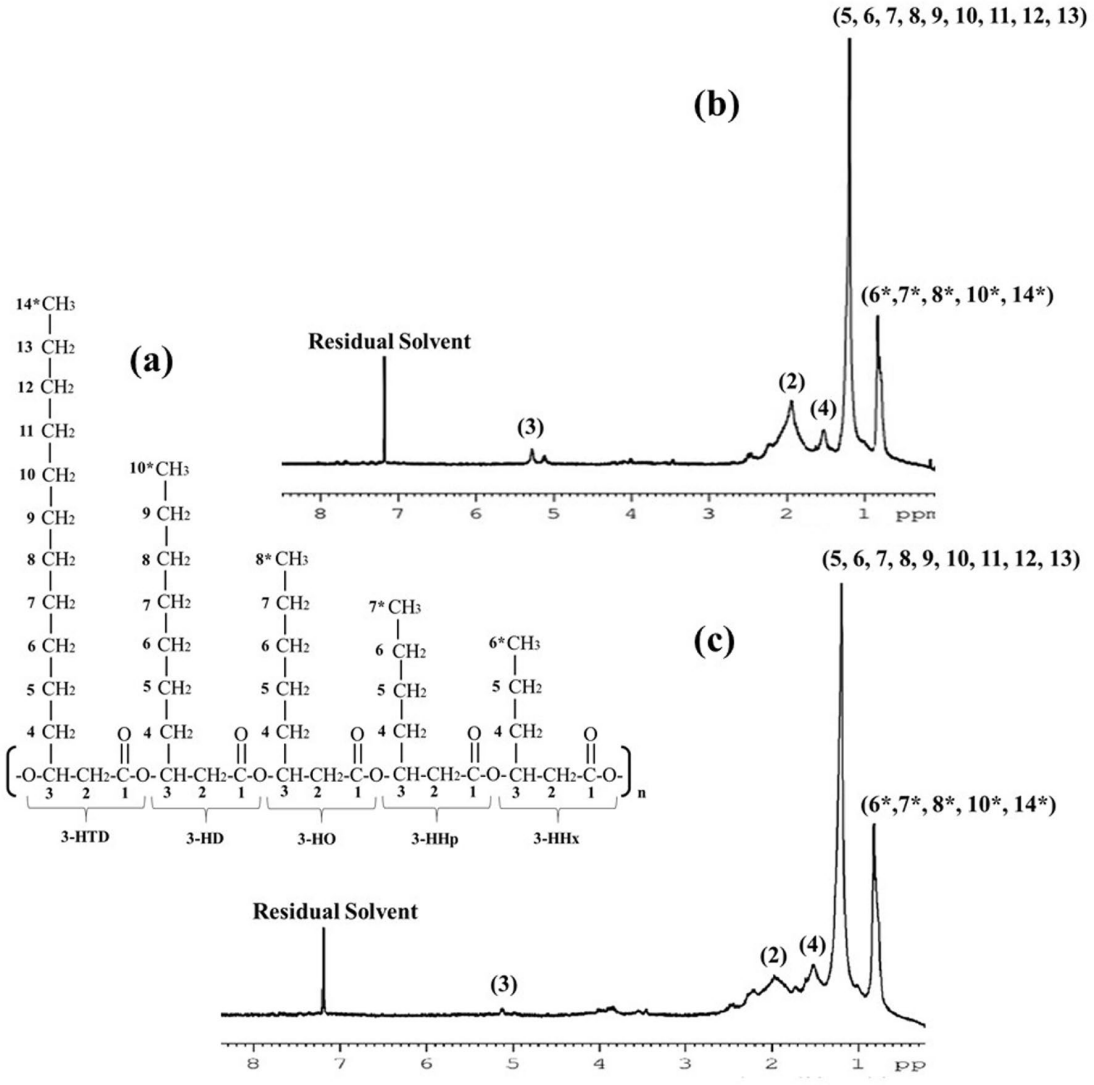
Proton NMR spectroscopic analysis of the polymer produced by *Pseudomonas aeruginosa* MCC 5300 in TSB media at various growth conditions at 24 h of incubation. (a) Structure of mcl‐PHA copolymer. (b) Proton NMR spectrum of the polymer produced in TSB media containing oleic acid. (c) Proton NMR spectrum of the polymer produced in TSB media containing oleic acid and 1 mM sodium azide.

The polymer produced by *P. aeruginosa* MCC 5300 in TSB media containing oleic acid with 1 mM sodium azide concentration was analysed using proton NMR spectroscopy using TSB media containing only oleic acid as a control and PHBV as a standard. The NMR spectrum of the polymer produced by bacteria in the presence of sodium azide revealed five prominent peaks, indicating the protons belonging to five different chemical environments. A peak in the 5.1–5.3 ppm range was observed, similar to the control and standard, indicating a highly de‐shielded proton of the –CH group bound to the hydroxyl group involved in ester linkage. Similarly, a peak in the range of 2–2.5 ppm indicates the presence of protons of the methylene group attached to the carbonyl carbon atom. The –CH group and methylene group form the fundamental backbone of PHA. The peaks observed at 1.5, 1.1 and 0.8 ppm indicate the protons of the methylene group bound to the β‐carbon atom, methylene groups attached to the fourth carbon atom and highly de‐shielded protons of terminal methyl groups, respectively. These three peaks indicate the alkyl side chain of the polymer. A similarity in the peaks obtained for the polymer produced by the bacteria with the standard suggests the polymer is PHA (Figures 10b,c and S3). A prominent peak at 1.1 ppm indicates the presence of multiple methylene groups attached to the fourth carbon atom (Figure 10b,c). Similarly, a prominent peak at 1.2 ppm was observed in mcl‐PHA copolymer obtained from *Pseudomonas rhizophila* S211 (Hammami et al. 2024). The similarity of peaks with the mcl‐PHA copolymer of *P. rhizophila* S211 and the FTIR spectrum in the present study confirms the polymer obtained from *P. aeruginosa* MCC 5300 as a mcl‐PHA. The similarity of peaks between the mcl‐PHA produced by bacteria in the presence of azide and mcl‐PHA of the control group shows the absence of any structural changes in mcl‐PHA monomers (Figure 10b,c).

## 4 Conclusion

*P. aeruginosa* MCC 5300 exhibited high growth and PHA production by utilising oleic acid in the media. The bacteria produced a high PHA yield of about 1.316 g/L with a PHA content of 39.4% CDW at a short duration of 24 h of growth in TSBOA. Adding sodium azide to the TSB media containing oleic acid increased PHA production in the bacteria. The PHA yield reached its maximum value of about 2.021 g/L with a PHA content of 66.4% CDW in TSB media containing oleic acid and 1 mM sodium azide concentration at 24 h of growth. The production of a high amount of PHA at a short duration of 24 h of growth in nutrient‐enriched media was not reported previously. The FTIR and proton NMR spectroscopy revealed the polymer as an mcl‐PHA copolymer. The reduced activity of cytochrome c oxidases by oleic acid shifted the electron flux from the cytochrome c pool to the ubiquinol pool, causing an increased activity of ubiquinol oxidases. To maintain the proton gradient, a high amount of NADH contributes electrons for the activity of ubiquinol oxidases, thus reducing its levels in the bacterial cells. The inhibition of bo_3_‐oxidase activity by azide ions resulted in the transition of electron flux originating from NADH to bd‐oxidase, further reducing the NADH levels to maintain the proton gradient for energy generation and bacterial growth. The excessive accumulation of mcl‐PHA in the bacterial cells during terminal oxidase‐inhibited conditions prevented the depletion of NADH levels in the bacterial cells by degradation of the polymer to yield PHA monomers that are utilised by the cells to generate NADH in the bacterial cells. Thus, mcl‐PHA maintains redox homeostasis in the *P. aeruginosa* MCC 5300 cells.

## Author Contributions

**Raghavendra Paduvari:** investigation, formal analysis, visualisation, writing – original draft. **Divyashree Somashekara:** conceptualization, supervision, validation, writing – review and editing, validation.

## Ethics Statement

The authors have nothing to report.

## Conflicts of Interest

The authors declare no conflicts of interest.

## Supporting information


**Figure S1:** Cell viability of *Pseudomonas aeruginosa* MCC 5300 at various concentrations of sodium azide (mM).
**Figure S2:** FTIR spectroscopic analysis of standard polyhydroxybutyrate‐co‐valerate (PHBV) copolymer.
**Figure S3:** Proton NMR spectroscopic analysis of standard PHBV. (a) Chemical structure of PHBV. (b) Proton NMR spectrum of standard PHBV.

## Data Availability

The data that support the findings of this study are available on request from the corresponding author. The data are not publicly available due to privacy or ethical restrictions.
