# Supplementary material for: A Novel Link Between Electron Transport Chain Modulation and mcl‐PHA Production in Pseudomonas aeruginosa 5300 Using Azide as a Modulator
Source: Environ Microbiol Rep. 2026 Jan 23;18(1):e70251. doi: 10.1111/1758-2229.70251 (PMC12829472; doi:10.1111/1758-2229.70251)
Supplement: Supplementary file 1 — Figure S1: Cell viability of Pseudomonas aeruginosa MCC 5300 at various concentrations of sodium azide (mM). Figure S2: FTIR spectroscopic analysis of standard polyhydroxybutyrate‐co‐valerate (PHBV) copolymer. Figure S3: Proton NMR spectroscopic analysis of standard PHBV. (a) Chemical structure of PHBV. (b) Proton NMR spectrum of standard PHBV. [file EMI4-18-e70251-s001.docx]

**Supplementary Information**

**A Novel Link Between Electron Transport Chain Modulation and mcl-PHA Production in *Pseudomonas aeruginosa* 5300 using Azide as a Modulator**

Raghavendra Paduvari and Divyashree Mysore Somashekara^*^

Department of Biotechnology, Manipal Institute of Technology, Manipal Academy of Higher Education, Manipal, 576104, Karnataka, India.

^*^Corresponding author: Email ID: [divyashree.ms@manipal.edu](mailto:divyashree.ms@manipal.edu)

1. **Materials and Methods**
   1. **Estimation of minimum inhibitory concentration (MIC)**
      1. **Preparation of standard bacterial culture for MIC**

A loopful of bacterial colony was inoculated into 5 mL nutrient broth taken in a test tube and incubated at 30 ºC, 180 rpm for 18 h. The bacterial cultures were centrifuged at 7781 xg for 8 min to collect the cell pellets. The cell pellets were suspended in sterile distilled water, and the optical density at 620 nm was adjusted to 1 with a viable cell number of 77×10^5^ CFU/mL (colony forming unit per millilitre)(Wiegand et al., 2008).

- - 1. **Preparation of sodium azide solution**

A stock solution of sodium azide (19.5 mg/mL) was made using sterile distilled water. The stock solution was added to the reaction mixture in a 96-well plate to give concentrations ranging from 0.5 mM to 30 mM in a 300 µL reaction mixture.

- - 1. **Resazurin microtitre assay**

The MIC of sodium azide for the bacterium was determined using a resazurin dye-based microtitre assay. An equal amount of standard bacterial culture was mixed with reaction mixture consisting of sodium azide dilutions, nutrient broth, and 0.675% resazurin dye to a 300 µL total volume in a 96-well plate. A control without sodium azide was included. The plate was incubated at 30 °C for 18 h, then centrifuged at 7781 xg for 8 min to separate cells from the supernatant. Absorbance (A) was measured at 570 nm and 600 nm for reduced and unreduced dye, respectively(Sarker et al., 2007). The percentage viability of bacterial cells was estimated as shown below.

$$Percentage viability=\frac{A 570 nm-A 600 nm (Test)}{A 570 nm-A 600 nm (Control)}\times100$$

1. **Results and discussion**
   1. **Concentration-dependent cell viability analysis of *Pseudomonas aeruginosa* MCC 5300 using sodium azide**

The resazurin dye-based microtitre assay was performed on *Pseudomonas aeruginosa* MCC 5300 at various sodium azide concentrations by taking bacterial culture without sodium azide as a control (0 mM). The increase in sodium azide concentration caused a decline in the percentage cell viability of the bacteria. This shows the growth-inhibitory nature of sodium azide at higher concentrations. The percentage cell viability was 106 ± 5.7 % and 99 ± 1.8 % at a concentration of 0.5 mM and 1 mM, respectively, similar to the control with a percentage cell viability of 100 ± 9.5 %. An increase in the sodium azide concentration from 1 mM to 3 mM caused a 24 % reduction in the percentage of cell viability. The percentage viability reduction shows that the MIC of sodium azide is 3 mM for *Pseudomonas aeruginosa* MCC 5300, below which there was no significant decrease in the percentage cell viability. Therefore, sodium azide concentration range below 3 mM was taken to analyze its effect on bacterial growth and PHA production.

**
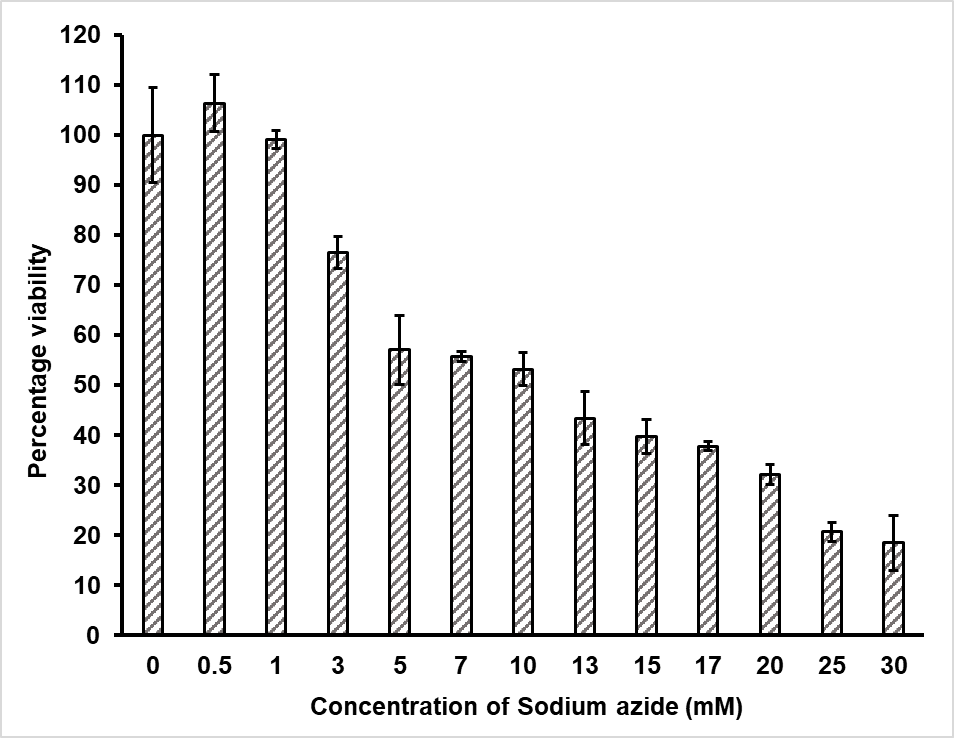
**

**Figure S1:** Cell viability of Pseudomonas aeruginosa MCC 5300 at various concentrations of sodium azide (mM).

- 1. **FTIR spectrum of the standard polyhydroxybutyrate-co-valerate (PHBV)**


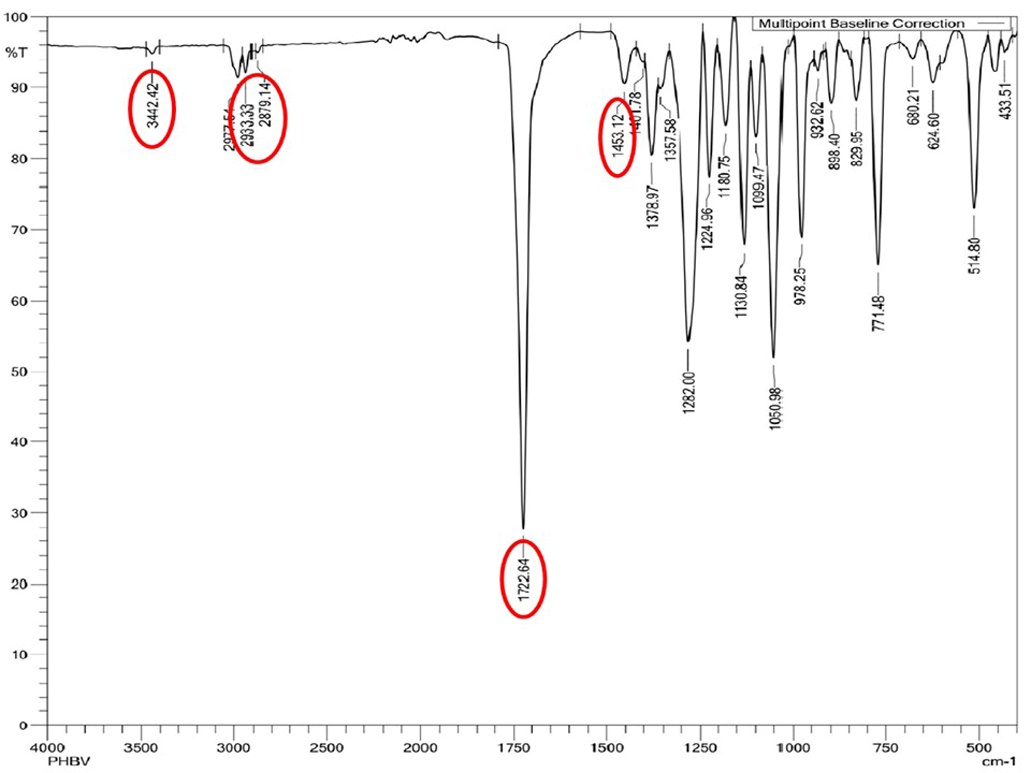


**Figure S2:** FTIR spectroscopic analysis of standard polyhydroxybutyrate-co-valerate (PHBV) copolymer.

- 1. **Proton NMR spectrum of the standard polyhydroxybutyrate-co-valerate (PHBV)**


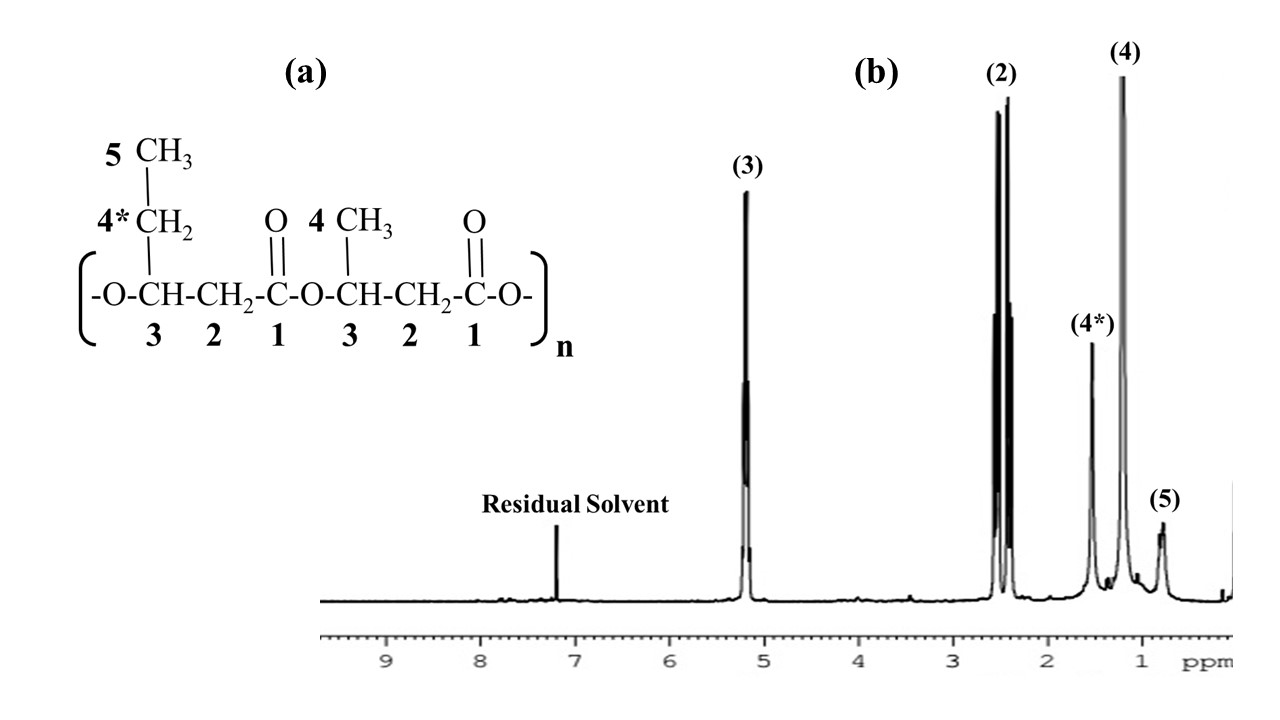


**Figure S3:** Proton NMR spectroscopic analysis of standard PHBV. **(a)** Chemical structure of PHBV. **(b)** Proton NMR spectrum of standard PHBV.

1. **References**

Sarker, S.D., Nahar, L., Kumarasamy, Y., 2007. Microtitre plate-based antibacterial assay incorporating resazurin as an indicator of cell growth, and its application in the in vitro antibacterial screening of phytochemicals. Methods 42, 321–324. https://doi.org/10.1016/j.ymeth.2007.01.006

Wiegand, I., Hilpert, K., Hancock, R.E.W., 2008. Agar and broth dilution methods to determine the minimal inhibitory concentration (MIC) of antimicrobial substances. Nat. Protoc. 3, 163–75. https://doi.org/10.1038/nprot.2007.521
